# Cortical axonal loss is associated with both gray matter demyelination and white matter tract pathology in progressive multiple sclerosis: Evidence from a combined MRI-histopathology study

**DOI:** 10.1177/1352458520918978

**Published:** 2020-05-11

**Authors:** Svenja Kiljan, Paolo Preziosa, Laura E Jonkman, Wilma DJ van de Berg, Jos Twisk, Petra JW Pouwels, Geert J Schenk, Maria A Rocca, Massimo Filippi, Jeroen JG Geurts, Martijn D Steenwijk

**Affiliations:** Department of Anatomy & Neurosciences, Amsterdam UMC, locatie VU University Medical Center, Amsterdam, The Netherlands/Amsterdam Neuroscience, MS Center Amsterdam, Amsterdam, The Netherlands; Department of Anatomy & Neurosciences, Amsterdam UMC, locatie VU University Medical Center, Amsterdam, The Netherlands/Amsterdam Neuroscience, MS Center Amsterdam, Amsterdam, The Netherlands/Neuroimaging Research Unit, Institute of Experimental Neurology, Division of Neuroscience, and Neurology Unit, IRCCS San Raffaele Scientific Institute, Milan, Italy; Department of Anatomy & Neurosciences, Amsterdam UMC, locatie VU University Medical Center, Amsterdam, The Netherlands; Department of Anatomy & Neurosciences, Amsterdam UMC, locatie VU University Medical Center, Amsterdam, The Netherlands; Department of Epidemiology and Biostatistics, Amsterdam UMC, locatie VU University Medical Center, Amsterdam, The Netherlands; Department of Radiology and Nuclear Medicine, Amsterdam Neuroscience, MS Center Amsterdam, Amsterdam UMC, locatie VU University Medical Center, Amsterdam, The Netherlands; Department of Anatomy & Neurosciences, Amsterdam UMC, locatie VU University Medical Center, Amsterdam, The Netherlands/Amsterdam Neuroscience, MS Center Amsterdam, Amsterdam, The Netherlands; Neuroimaging Research Unit, Institute of Experimental Neurology, Division of Neuroscience, and Neurology Unit, IRCCS San Raffaele Scientific Institute, Milan, Italy; Neuroimaging Research Unit, Institute of Experimental Neurology, Division of Neuroscience, Neurology Unit, Neurophysiology Unit, IRCCS San Raffaele Scientific Institute, Milan, Italy/Vita-Salute San Raffaele University, Milan, Italy; Department of Anatomy & Neurosciences, Amsterdam UMC, locatie VU University Medical Center, Amsterdam, The Netherlands/Amsterdam Neuroscience, MS Center Amsterdam, Amsterdam, The Netherlands; Department of Anatomy & Neurosciences, Amsterdam UMC, locatie VU University Medical Center, Amsterdam, The Netherlands/Amsterdam Neuroscience, MS Center Amsterdam, Amsterdam, The Netherlands

**Keywords:** Multiple sclerosis, magnetic resonance imaging, axonal loss, histopathology, post mortem, progressive

## Abstract

**Background:**

Neuroaxonal degeneration is one of the hallmarks of clinical deterioration in progressive multiple sclerosis (PMS).

**Objective:**

To elucidate the association between neuroaxonal degeneration and both local cortical and connected white matter (WM) tract pathology in PMS.

**Methods:**

*Post-mortem in situ* 3T magnetic resonance imaging (MRI) and cortical tissue blocks were collected from 16 PMS donors and 10 controls. Cortical neuroaxonal, myelin, and microglia densities were quantified histopathologically. From diffusion tensor MRI, fractional anisotropy, axial diffusivity (AD), radial diffusivity (RD), and mean diffusivity (MD) were quantified in normal-appearing white matter (NAWM) and white matter lesions (WML) of WM tracts connected to dissected cortical regions. Between-group differences and within-group associations were investigated through linear mixed models.

**Results:**

The PMS donors displayed significant axonal loss in both demyelinated and normal-appearing (NA) cortices (*p* < 0.001 and *p* = 0.02) compared with controls. In PMS, cortical axonal density was associated with WML MD and AD (*p* = 0.003; *p* = 0.02, respectively), and NAWM MD and AD (*p* = 0.04; *p* = 0.049, respectively). NAWM AD and WML AD explained 12.6% and 22.6%, respectively, of axonal density variance in NA cortex. Additional axonal loss in demyelinated cortex was associated with cortical demyelination severity (*p* = 0.002), explaining 34.4% of axonal loss variance.

**Conclusion:**

Reduced integrity of connected WM tracts and cortical demyelination both contribute to cortical axonal loss in PMS.

## Introduction

Multiple sclerosis (MS) is a disease of the central nervous system, characterized by inflammation, demyelination, and neurodegeneration of both the white matter (WM) and the gray matter (GM). Effective—mainly anti-inflammatory—treatments have become increasingly available for relapsing remitting (RR) MS. Although ocrelizumab and siponimod have recently been approved as treatments for progressive multiple sclerosis (PMS), treatment options that limit neurodegeneration are still scarce and strongly needed.^1^ Given the more severe clinical deficits in PMS, it is of utmost importance to unravel the underlying pathological substrate. While inflammatory white matter lesions (WML) are the pathological hallmark of (early) MS, neuroaxonal degeneration is the most characteristic pathological finding in PMS.^2^ This neuroaxonal degeneration can be detected as atrophy on magnetic resonance imaging (MRI),^3^ and the rate of GM atrophy has been shown to increase with disease progression^4^ and is especially relevant for clinical decline in PMS.^5,6^

Several candidate mechanisms underlying neuroaxonal degeneration have been hypothesized and empirically tested.^7^ In early primary PMS, degeneration of WM tracts measured with MRI has been shown to precede atrophy in connected GM regions, suggesting that neuroaxonal loss in GM may be secondary to WM tract degeneration, indicating Wallerian degeneration as an underlying mechanism.^8^ In line with this, a *post-mortem* study found retrograde neuroaxonal degeneration in cortex close to a WML.^9^ However, in long-standing disease, connected WM pathology could explain cortical GM atrophy only in relapsing remitting multiple sclerosis (RRMS) patients and not in PMS patients, suggesting that cortical GM atrophy and WM damage are (at least partly) independent disease processes in PMS.^10^ Local pathological processes in cortical GM and meninges may be partly responsible for the uncoupling between WM and GM pathology in long-standing MS, through their direct effects on cortical axons and neurons. For example, histopathological studies show that compared with RRMS, PMS shows extensive subpial demyelination and meningeal inflammation, processes that both have been associated with neuritic loss.^11^ Yet, to what extent “local” cortical damage and “remote” WM damage contribute to cortical neuroaxonal degeneration in PMS is still unknown.

Therefore, we set up a combined MRI and histopathology study to investigate the relationship of local cortical GM damage and remote WM damage (in connected tracts) to cortical neuroaxonal loss in PMS. Cortical neuroaxonal degeneration and local cortical pathology were quantified in using histology, while *post-mortem* MRI was used to quantify damage in WM tracts connected to the corresponding cortex.

## Methods

### Subjects and data collection pipeline

In this study, 16 PMS cases with clinically definite and neuropathologically verified MS and 10 non-neurological controls were included shortly after death (Table 1; supplementary material). The 3T MRI of the brain *in situ* was performed, followed by tissue dissection during autopsy. The brain was cut into 10-mm-thick coronal brain slices. Systematic dissection of the following six cortical regions was performed: the superior and inferior frontal gyrus, the anterior and posterior cingulate cortex, the superior temporal gyrus, and the inferior parietal lobule.^12^ In total, 110 paraffin-embedded tissue blocks were collected. Data were collected in collaboration with the Netherlands Brain Bank (NBB; http://www.brainbank.nl) and Normal Aging Brain Collection Amsterdam^13^ (NABCA; http://www.nabca.eu). This study complies with the institutional ethics guidelines. Subjects or their next of kin provided written informed consent for the use of their tissue and clinical information for research purposes to the NBB or NABCA.

**Table 1.** Demographic and clinical data of subjects.

Case	Sex	Age of death (y)	Age of diagnosis (y)	Disease duration (y)	MS type	*Post-mortem* delay to autopsy (h:m)	Cause of death	Number of available tissue blocks
MS								
1	M	66	41	25	PPMS	8:55	Euthanasia	5
2	M	62	21	41	SPMS	9:20	Euthanasia	3
3	F	52	21	31	PPMS	7:40	Euthanasia	1
4	M	51	30	21	SPMS	9:40	Euthanasia	4
5	M	83	38	45	SPMS	7:00	Pneumonia	5
6	F	63	30	33	SPMS	7:20	Euthanasia	1
7	F	40	32	8	SPMS	7:10	Ileus	1
8	F	71	39	32	SPMS	7:55	Euthanasia	5
9	F	57	28	29	SPMS	8:55	Euthanasia	5
10	F	49	49	25	SPMS	7:45	Pneumonia	3
11	F	83	23	60	SPMS	7:40	Euthanasia	4
12	F	62	39	23	SPMS	8:45	Euthanasia	4
13	F	65	25	40	SPMS	9:40	Sepsis	3
14	F	77	51	26	PPMS	8:35	Colorectal cancer	5
15	F	81	30	51	SPMS	6:10	Respiratory insufficiency	2
16	M	76	34	42	PPMS	8:30	Euthanasia	3
Controls								
1	M	77				11:35	Pneumonia	6
2	F	69				13:00	Pulmonary embolism	6
3	M	59				8:00	Euthanasia	6
4	F	71				6:50	Lung cancer	6
5	M	74				16:00	Colorectal cancer	6
6	M	72				11:45	Esophageal cancer	6
7	F	79				6:15	Unknown	6
8	F	78				10:00	Unknown	5
9	F	72				7:15	Heart failure	6
10	M	74				10:15	Euthanasia	2

MS: multiple sclerosis; y: years; h: hours; m: minutes; M: male; F: female; PPMS: primary progressive multiple sclerosis; SPMS: secondary progressive multiple sclerosis.

### Immunohistochemistry and Bielschowsky staining

**Figure 1.**
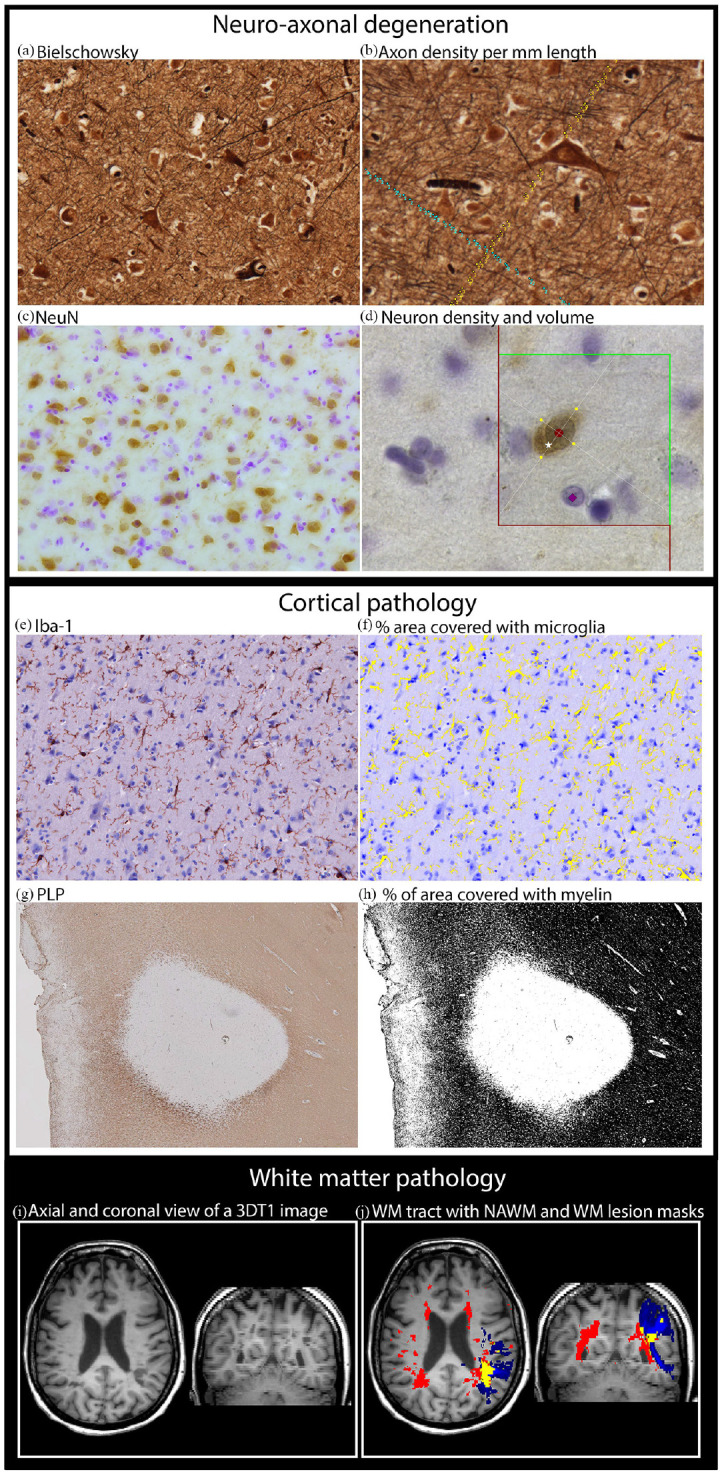
Study methods. (a) Cortical axons (Bielschowsky staining). (b) Quantification of cortical axonal density was performed by counting parallel and perpendicular axons, to be unbiased for fiber orientation. Parallel and perpendicular axons were counted when intersecting with the green line (either counted with blue or yellow dots). (c) Cortical neurons (NeuN staining). (d) Quantification of neuron density was performed using the optical fractionator, and an example of neuron that was counted is indicated by white star. Quantification of neuronal volume was performed using the nucleator, where red circle indicates the center of the neuron while four yellow dots indicate the outer cell surface of the neuron and intersection with the four lines originating from the neuronal center. The counterstaining (Nissl) is visible indicated by purple square. (e) Cortical microglia (Iba-1 staining). (f) Quantification of microglia density, based on a mask of Iba-1 staining (yellow). (g) Myelin (PLP staining). (h) Quantification of myelin density, based on a mask of PLP staining (black). (i) 3DT1 MRI image. (j) Example of a white matter (WM) tract in blue, a whole brain WM lesion mask in red, and their overlap in yellow.

Immunostaining of cortical myelin and microglia, as well as a Bielschowsky silver staining to visualize axons, was performed on consecutive 10-µm-thick sections. Immunostaining of neurons counterstained with Nissl was performed on 20-µm-thick sections (Figure 1(a)–(h) shows an overview of these stainings and their analysis). Detailed staining procedures can be found in the supplementary material and elsewhere.^14^

### Quantification of neuroaxonal degeneration and cortical pathology

Neuroaxonal degeneration and cortical pathology were quantified in lesioned or cortical non-appearing gray matter (NAGM) in six-layered and non-curved parts of the cortex. Cortex was classified as lesioned or NAGM by two independent raters (S.K. and P.P.) based on myelin immunostaining. Lesions were defined as cortical areas with a complete lack of myelination.

Images of Bielschowsky staining were acquired at 200× magnification. Analyses of axonal density were performed manually in ImageJ/Fiji (version 1.52a, https://imagej.net/Fiji).^15^ Axonal density was obtained by counting the intersection of axons with a vertical line running from the pial surface to the WM and with horizontal lines perpendicular to the vertical line (Figure 1(b)). Axonal density was expressed as axon number per millimeter. Neuronal density and volume were visualized at 630× magnification and quantified using Stereoinvestigator software, specifically the optical fractionator tool for neuronal counts and the nucleator tool for neuronal volume (Figure 1(d)).

Microglia density was quantified using a microscope with a spectral imaging device; images were acquired at 200× magnification. Spectral information of Iba-1 immunostaining was used to compute a corresponding mask, and microglia density was expressed as percentage of stained area of the image area (Figure 1(f)).

Myelin density was imaged at 50× magnification. A mask of the myelin staining was computed using ImageJ, and myelin density was expressed as percentage of stained area (Figure 1(h)). Additional methodological details about the quantification process can be found in the supplementary material and elsewhere.^14^

### Quantification of WM pathology in connected tracts

The MRI of the brain *in situ* was acquired using a 3T whole-body scanner and an eight-channel phased-array head coil. The protocol included a three-dimensional T1-weighted fast spoiled gradient-echo sequence for volumetric analysis, a three-dimensional fluid-attenuated inversion-recovery sequence for WML detection, and a diffusion-tensor imaging sequence for tractography. Prior to analysis, three-dimensional images were corrected for geometrical distortions due to gradient nonlinearity.

An atlas of WM connections running from the six cortical regions (dissected during autopsy) to other GM regions was constructed in an independent cohort of 60 healthy controls to overcome the potentially confounding effect of MS-related WML on tractography methods. This cohort was scanned *in vivo* using the same system and protocol as for *post-mortem* acquisitions. Diffusion-weighted images were corrected for motion and eddy current distortion using FMRIB’s Diffusion Toolbox (part of FSL 5.0.9 https://fsl.fmrib.ox.ac.uk/).^16^ Probabilistic tractography was performed, and probabilistic maps were binarized at 0.25% of the total number of generated streamlines.^17^ A group-level probabilistic WM tract atlas was finally constructed.

The atlas was used to quantify WM pathology in the donors. The WML were automatically segmented using an in-house developed algorithm and manually adjusted by an experienced rater (P.P.), followed by WML filling. Overlap between the WM tract atlas and the WML mask was used to determine the percentage of WML volume per WM tract. For each subject, the weighted average of fractional anisotropy (FA), mean diffusivity (MD), radial diffusivity (RD), and axial diffusivity (AD) were determined within normal-appearing white matter (NAWM) and WML per tract (Figure 1(i) and (j)). Segmented WM tracts were visually inspected, as well as the overlap between the dissected tissue blocks and GM mask seed regions to reassure accuracy. Additional methodological details about MRI analysis and acquisition can be found in the supplementary material.

### Statistical analysis

SPSS (version 22; IBM, Chicago, IL, USA) was used to perform statistical analyses. To account for interdependences of tissue blocks within subjects, linear mixed models were used to evaluate between-group differences and within-group relations using age, gender, and *post-mortem* delay (PMD) as nuisance covariates. Values of *p* < 0.05 were considered significant. Explained variance was calculated as follows: the reduction in residual variance of the model including the predictor of interest (e.g. cortical demyelination, WM damage, or microglia activation) compared with the model with only nuisance covariates (age, gender, and PMD) was expressed as a percentage and referred to as the explained variance.

The following evaluations were performed:

1. Group differences in measures of cortical neuroaxonal degeneration, demyelination, and microglia density between region of interests (ROIs) with MS cortical normal-appearing gray matter (NAGM), ROIs with MS lesioned cortex, and cortical ROIs from controls (*n* = 16 patients with MS, 54 tissue blocks, 76 ROIs; 49 ROIs with cortical NAGM, and 27 ROIs with lesioned cortex; *n* = 10 controls subjects, 56 tissue blocks, and 56 ROIs with cortical GM).
2. Group differences in measures of diffusion MRI were evaluated between MS NAWM and control NAWM and between MS NAWM and MS WML (*n* = 16 patients with MS, 54 WM tracts connected to dissected GM regions, and *n* = 10 controls, 56 WM tracts connected to dissected GM regions).

Then, tissue blocks from MS patients having both an ROI with cortical NAGM and an ROI with lesioned cortex were selected for further analysis (*n* = 11 MS patients, 22 tissue blocks, 44 ROIs; 22 ROIs with cortical NAGM and 22 ROIs with lesioned cortex). Then, we evaluated the following:

1. The relationship between axonal density in cortical NAGM and integrity measures of connected WM tracts was assessed in both MS patients and controls (*n* = 10 control subjects, 56 tissue blocks, and 56 ROIs with cortical GM).
2. In MS patients, the percentage change in axonal density and myelin density in the ROI with MS lesioned cortex was calculated compared with the ROI with MS cortical NAGM. The relationship between the percentage reduction in axonal density and percentage reduction in myelin density was assessed.
3. The association between cortical microglia density and axonal density in cortical NAGM and in lesioned cortex was tested in MS.

## Results

### Histopathology-measured cortical neuroaxonal degeneration

**Figure 2.**
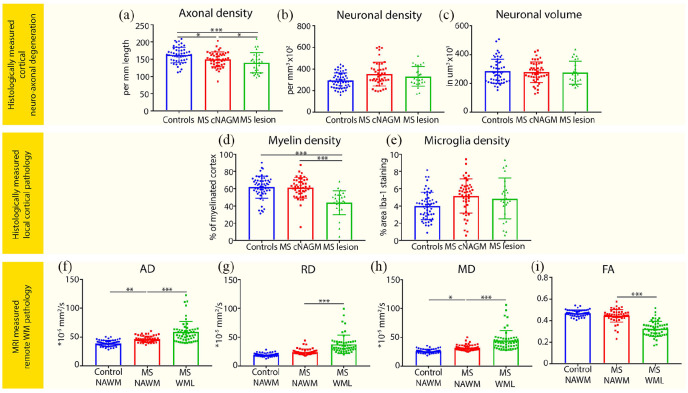
Axonal loss, cortical pathology, and WM pathology in multiple sclerosis. The first two rows show bar plots displaying group differences between controls, MS cortical normal-appearing gray matter (cNAGM), and MS lesioned cortex (MS lesion) on (a) axonal density, (b) neuronal density, (c) neuronal volume, (d) myelin density, and (e) microglia density. The bottom row shows bar plots displaying group differences between control normal-appearing white matter (NAWM), MS NAWM, and MS white matter lesions (WML) on (f) axial diffusivity (AD), (g) radial diffusivity (RD), (h) mean diffusivity (MD), and (i) Fractional anisotropy (FA). Dots represent data points of tissue blocks of subjects. *p < 0.05; **p ⩽ 0.01; ***p ⩽ 0.001.

**Figure 3.**
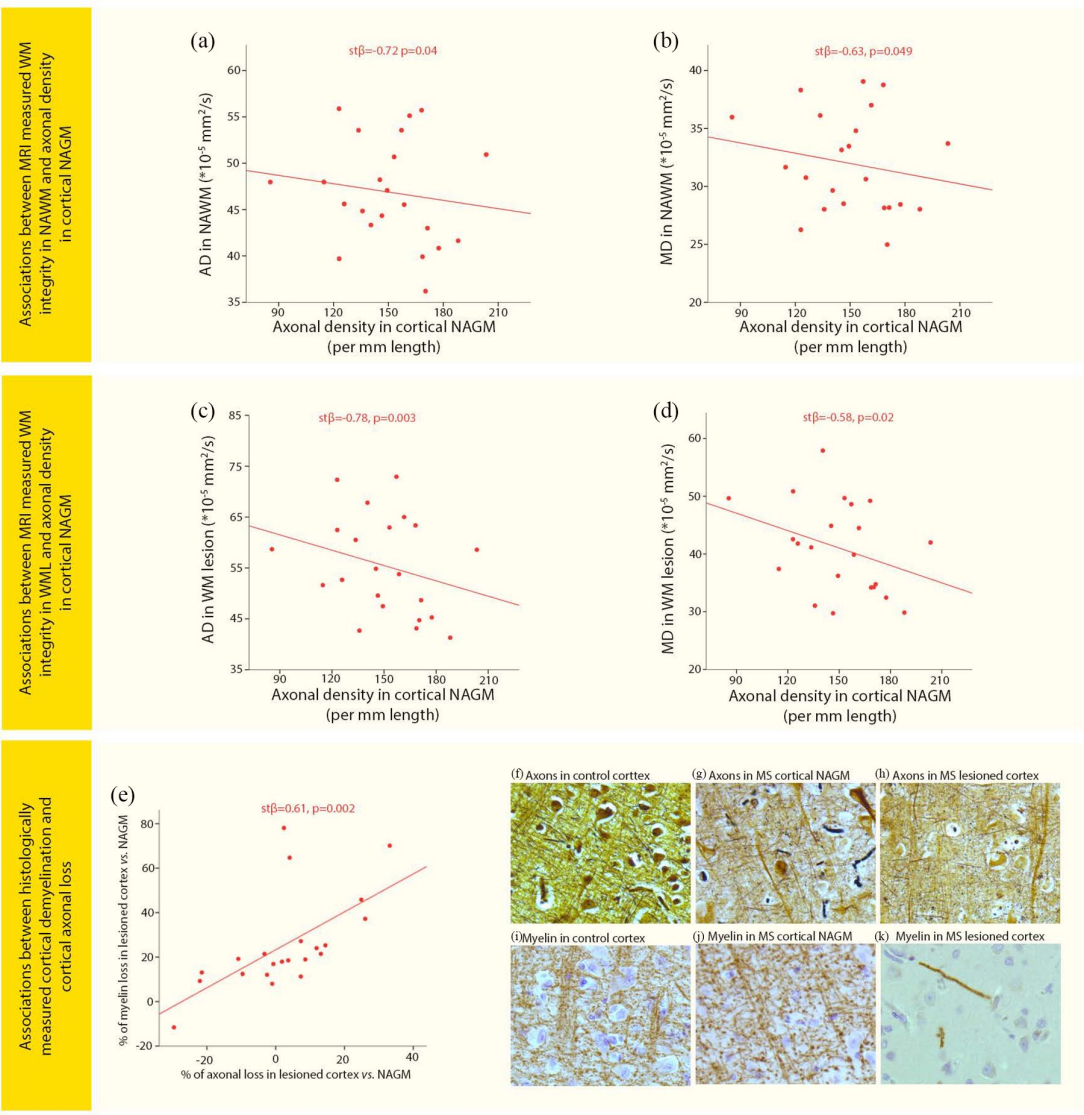
Associations of WM pathology and cortical pathology with cortical axonal loss. Scatter plots displaying associations between (a) axial diffusivity (AD) in normal-appearing white matter (NAWM) and axonal density in cortical normal-appearing gray matter (NAGM) in MS, (b) Mean diffusivity (MD) in NAWM and axonal density in cortical NAGM in MS, (c) AD in white matter lesions (WML) and axonal density in cortical NAGM in MS, (d) MD in WML and axonal density in cortical NAGM in MS, (e) the percentage reduction in axonal density in lesioned cortex compared with cortical NAGM and percentage reduction in myelin density in lesioned cortex compared with cortical NAGM. Images show axonal staining (Bielschowsky) in (f) control cortex, (g) MS cortical NAGM, and (h) MS lesioned cortex. Images showing myelin staining (PLP) in an adjacent tissue section and same location as the axonal pictures were taken in (i) control cortex, (j) MS cortical NAGM, and (k) MS lesioned cortex.

Compared with controls, a reduction in axonal density was observed in MS cortical NAGM (on average: –9.2%) and lesioned cortex (on average: –17.4%; *p* = 0.02 and *p* < 0.001, respectively; Figure 2(a); Table 2). In addition, reduced axonal density in MS lesioned cortex compared with MS NAGM was found (*p* = 0.02; Figure 2(a)). No group differences were observed in cortical neuronal density or volume (Figure 3(b) and (c); Table 2).

**Table 2.** Group differences in cortical neuroaxonal measures, myelin density, and microglia density.

	MS (*N* = 54)		Controls (*N* = 56)	Group differences	MS cortical NAGM vs control cortical GM		MS demyelinated cortex vs control cortical GM		MS cortical NAGM vs MS demyelinated cortex	
	Cortical NAGM	Demyelinated cortex	Cortical GM	*F, p*	Estimated mean difference (95% CI)	*p*	Estimated mean difference (95% CI)	*p*	Estimated mean difference (95% CI)	*p*
	Mean (SE)	Mean (SE)	Mean (SE)							
Axonal density (axons/mm)	148.83 (4.07)	135.47 (5.25)	164.20 (4.22)	7.92, 0.001	−15.37 (–27.99 to 2.75)	0.02	−28.72 (–43.17 to 14.28)	<0.001	13.35 (2.07 to 24.63)	0.02
Neuronal density (neurons/mm^2^)	3.49 × 10^4^ (0.21 × 10^4^)	30472.39 (2428.09)	31475.46 (2321.15)	0.89, 0.42	n.s. at group level	n.s. at group level	n.s. at group level	n.s. at group level	n.s. at group level	n.s. at group level
Neuronal volume (µm^3^)	2740.46 (130.69)	2802.89 (176.77)	2814.21 (128.98)	0.08, 0.92	n.s. at group level	n.s. at group level	n.s. at group level	n.s. at group level	n.s. at group level	n.s. at group level
Myelin density (%)	61.41 (2.30)	42.99 (2.89)	62.97 (2.39)	20.57, <0.001	−1.56 (–8.71 to 5.60)	0.66	−19.98 (–28.05 to 11.91)	<0.001	18.42 (12.45 to 24.40)	<0.001
Microglia density (%)	4.54 (0.49)	4.78 (0.54)	4.01 (0.58)	0.64, 0.53	n.s. at group level	n.s. at group level	n.s. at group level	n.s. at group level	n.s. at group level	n.s. at group level

n.s.: not significant; N: number of tissue blocks; MS: multiple sclerosis; NAGM: normal-appearing gray matter; GM: gray matter; SE: standard error; CI: confidence interval.

In this table, the mean and standard error of histological variables are displayed for controls and MS patients. Only variables that significantly differed at group level were further explored post hoc. Statistically significant comparisons are underlined.

### Histopathology-measured cortical GM pathology

MS lesioned cortex displayed lower myelin density compared with MS NAGM and control GM (both *p* < 0.001), while MS NAGM myelin density did not show differences compared with control GM (Figure 2(d); Table 2). No differences in microglia density were found between MS and controls, possibly as a result of high variability in the measurements (Figure 2(e); Table 2).

### MRI measured connected WM pathology

The whole-brain WML volume in MS patients was 47.5 ± 24.5 mL and spanned 3.54% ± 4.03% of the measured WM tract volume. Compared with connected NAWM of controls, MS patients showed increased AD and MD (*p* = 0.01; *p* = 0.03, respectively), but no difference in FA and RD. In MS WML compared with MS NAWM, AD, RD, and MD were increased and FA was decreased (all *p* < 0.001; Figure 3(f)–(i); Table 3).

**Table 3.** Group differences in WM tract integrity measures of FA, AD, RD, and MD.

	MS (*N* = 54)		Controls (*N* = 56)	MS NAWM vs Control NAWM		MS NAWM vs MS WML	
	NAWM	WML	NAWM				
	Mean (SE)	Mean (SE)	Mean (SE)	Estimated mean difference (95% CI)	*p*	Estimated mean difference (95% CI)	*p*
AD (×10^–5^ mm^2^/s)	46.3 (0.74)	58.7 (2.56)	38.9 (0.70)	5.39 (1.17 to 9.60)	0.01	12.4 (85.9 to 16.1)	<0.001
RD (×10^–5^ mm^2^/s)	24.3 (0.68)	37.5 (2.20)	19.5 (0.39)	3.22 (–0.36 to 6.80)	0.08	13.1 (10.2 to 16.0)	<0.001
MD (×10^–5^ mm^2^/s)	31.7 (0.67)	44.6 (2.30)	26.0 (0.47)	3.99 (0.40 to 7.75)	0.03	12.8 (9.65 to 15.9)	<0.001
FA	0.44 (7.79 × 10^–3^)	0.33 (9.45 × 10^–3^)	0.47 (3.85 × 10^–3^)	−0.01 (–0.06 to 0.03)	0.58	−0.12 (–0.14 to 0.10)	<0.001

MS: multiple sclerosis; NAWM: normal-appearing white matter; WML: white matter lesions; SE: standard error; CI: confidence interval; AD: axial diffusivity; RD: radial diffusivity; MD: mean diffusivity; FA: fractional anisotropy. Statistically significant comparisons are underlined.

### Associations between WM damage and axonal loss in normal-appearing cortical GM

In MS patients, AD_NAWM_ and MD_NAWM_ showed negative associations with axonal density of connected NAGM (*p* = 0.04 and *p* = 0.049, respectively), while RD_NAWM_ showed a trend (*p* = 0.06) and FA_NAWM_ was not associated (Figure 3(a) and (b); Table 4). Similar relationships were observed for within-WML diffusion measures, AD_WML_ and MD_WML_ showing negative associations with axonal density of connected NAGM (*p* = 0.003 and *p* = 0.02, respectively), while RD_WML_ and FA_WML_ were not associated (Figure 3(c) and (d); Table 4). In controls, none of the NAWM diffusivity measures was associated with axonal density of the connected cortical GM. The percentage of WML volume in tracts was not associated with axonal density in connected NAGM in MS patients.

**Table 4.** Associations between WM tract integrity measures or cortical pathology measures and cortical axonal loss.

	MS				Controls		
	stβ	*p*	95% CI	% of explained variance	stβ	*p*	95% CI
FA_NAWM_ × axonal density in cortical NAGM	−0.01	0.95	−0.47 to 0.44		0.05	0.80	−0.33 to 0.42
AD_NAWM_ × axonal density in cortical NAGM	−0.72	0.04	−1.39 to 0.05	12.6	0.07	0.68	−0.27 to 0.40
RD_NAWM_ × axonal density in cortical NAGM	−0.55	0.06	−1.12 to 0.02		0.14	0.48	−0.25 to 0.52
MD_NAWM_ × axonal density in cortical NAGM	−0.63	0.049	−1.26 to 0.005	7.1	0.12	0.51	−0.25 to 0.49
FA_WML_ × axonal density in cortical NAGM	−0.16	0.53	−0.68 to 0.36				
AD_WML_ × axonal density in cortical NAGM	−0.78	0.003	−1.25 to 0.31	22.6			
RD_WML_ × axonal density in cortical NAGM	−0.43	0.09	−0.92 to 0.07				
MD_WML_ × axonal density in cortical NAGM	−0.58	0.02	−1.04 to 0.11	4.6			
Cortical demyelination × axonal loss in lesioned cortex	0.61	0.002	0.23 to 0.98	34.4			
Microglia density × axonal density in cortical NAGM	0.08	0.78	−0.50 to 0.65				
Microglia density × axonal density in lesioned cortex	−0.25	0.39	−0.83 to 0.34				

MS: multiple sclerosis; NAWM: normal-appearing white matter; WML: white matter lesions; NAGM: normal-appearing gray matter; SE: standard error; CI: confidence interval; FA: fractional anisotropy; AD: axial diffusivity; RD: radial diffusivity; MD: mean diffusivity; stβ: standardized beta. Statistically significant comparisons are underlined.

AD in WML and NAWM explains the highest percentage of variance in axonal density in connected cortical NAGM, respectively, 22.6% and 12.6%. MD in WML explained 4.6% and MD in NAWM explained 7.1% of variance in axonal density in connected cortical NAGM (Table 4).

### Association between cortical axonal loss and local cortical pathology in MS

Additional axonal loss in lesioned cortex compared with cortical NAGM was significantly associated with the extent of cortical demyelination (*p* = 0.002; Figure 3(e)–(k)), while microglia density was not associated with axonal density in cortical NAGM or lesioned cortex. Cortical demyelination explained 34.4% of the variance in cortical axonal loss (Table 4).

## Discussion

In this study, we investigated the relationship between local cortical damage and remote WM damage (in connected tracts) to cortical neuroaxonal loss in PMS. Our results show that decreased WM tract integrity is related to axonal loss in connected cortical NAGM in PMS. AD in WML and in NAWM explained, respectively, 22.6% and 12.6% of the variance in axonal density in cortical NAGM in PMS. Furthermore, local cortical demyelination was associated with additional axonal loss in lesioned cortex and explained 34.4% of variance in axonal loss, while microglia showed no association.

We found cortical axonal, but not neuronal, loss in our cohort of PMS patients. Decreases in axonal density have consistently been reported in both WM and GM in (early) MS and related to disease progression.^18–22^ The results regarding neuronal loss are more divergent than axonal loss.^19,23–25^ In this study, neuronal density and volume were quantified considering all types of neurons in all cortical layers, differently from some other studies.^25^ Other factors that may explain the discrepancies in the literature include presence or absence of GM lesions and their types,^25^ whether tissue injury was profound or mild,^24^ presence or absence of meningeal B-cell follicles,^11^ and whether neuronal loss was measured in lesion enriched cortex or only within cortical lesions.^19^ Finally, tissue compaction can occur when neuropil degenerates, and this could mask neuronal loss.^26^

Diffusion-weighted imaging confirmed the presence of WM microstructural abnormalities in MS patients compared with controls. We detected increased AD and MD in MS NAWM compared with control NAWM, indicating axonal and myelin loss.^27,28^ In addition, these integrity changes were more pronounced in WML. Unaltered RD in NAWM compared with controls indicated relatively preserved myelin sheets in NAWM, while demyelination did occur in MS WML compared with MS NAWM.^29^ Of course, these imaging measures have caveats,^30^ and probably MD, AD, and RD are not entirely specific to their associated substrates. Our data suggest that the severity of axonal loss in MS WML and in NAWM is related to axonal loss in connected cortical NAGM. This is in line with several previous studies showing a relationship between GM atrophy and WM integrity, suggesting axonal dying back as an underlying process.^8,10^ Similar findings were also presented based on histology, highlighting a tract-specific relationship between cortical neurodegeneration and diffuse myelination changes in connected NAWM.^31^ In this particular study, neurodegeneration was measured as neuronal loss in cortical layer IV and as cortical thickness.^31^ We did find a similar relationship with cortical axonal loss in our study, which complements their results. Comparing, the lack of association with neuronal density in our study may be because of the layer-specific approach of the formerly mentioned study and the difference in histological methods applied to visualize neurons.

Our results show that WM integrity in MS WML, but not the volume of WML, within WM tracts is associated with axonal density in connected cortical NAGM. Accordingly, clinical findings show that not the volume of WM lesions but the diffusivity changes within lesions were related to clinical functioning.^32^ Also, compared with early MS so-called black holes on T1 are especially often observed in PMS. This type of WML is characterized by axonal loss.^33^ Thus, severity of WM damage, including chronic WML, increases over the disease course and may contribute to cortical axonal loss and dysfunction in PMS. Therefore, our results underline the clinical relevance of limiting WML origination and accumulation as much as possible in early disease to prevent accumulating cortical axonal loss in PMS.

Finally, we observed that cortical demyelination explains a portion of the variance in axonal density in PMS. This is in line with a previous study showing a higher number of transected axons in cortical lesions compared with cortical NAGM in MS.^24^ However, neuronal loss and also axonal loss have been shown to occur independent of demyelination to some extent.^19,23^ This discrepancy may be explained by this study having a larger sample size and using a different method of visualizing and measuring axons. We only find a relationship between axonal density and demyelination, but not with neuronal density. Explanations for this may be that transection of axons leads directly to axonal degeneration while neuronal degeneration only occurs at a later stage, and when neuropil including axonal arbors are degenerating rapidly, the remaining neurons may artificially seem like a higher density than they are in reality due to tissue compaction.^26^ Furthermore, microglia density was not associated with axonal loss in NAGM or lesioned cortex in our study. Although axonal loss in MS WML has consistently been related to inflammation in the cortex, neuroaxonal degeneration can also occur independently from inflammation.^19,20,24^ Summarizing, our study indicates that cortical demyelination but not microglia density contributes to axonal loss in the cortex of patients with PMS.

A particular asset of this study is the combination of two strong methodologies: histopathology, that is the gold standard technique to characterize cortical neurodegeneration, while diffusion MRI was used to quantify WM damage (which is not straight forward quantifiable in *post-mortem* human datasets). However, there were also some limitations. First, tracking of WM tracts may induce technical variability that may contribute to the moderately explained variance in our model. Also, death may influence the diffusivity properties of CSF and therefore our DTI-based findings. Recently was shown that post-mortem diffusivity measures were reduced compared with ante-mortem, possibly due to temperature changes of the deceased and decomposition and CSF absorption cessation.^34^ In this study *PMD* was used as a covariate in the analyses, to correct at least for a portion of above-mentioned effects of death on diffusivity. Furthermore, we analyzed axonal loss in lesion-enriched cortex instead of strictly lesioned cortex; this may lead to an underestimation axonal loss in cortical lesions. Finally, this study showed an association between cortical axonal density and both WM pathology and cortical demyelination, but what other processes can explain the remaining residual variance in cortical axonal density should be investigated in future research.

In conclusion, both local cortical demyelination and remote pathology in connected WM tracts are related to axonal loss in the cortex in PMS. In WML but also in NAWM, changes in AD, representing WM axonal loss, relate to the extent of axonal loss in the cortex. The extent of cortical demyelination, but not microglia density, also partly explains additional axonal loss in lesioned cortex in PMS.

## Supplemental Material

MSJ918978_supplemental_material – Supplemental material for Cortical axonal loss is associated with both gray matter demyelination and white matter tract pathology in progressive multiple sclerosis: Evidence from a combined MRI-histopathology study Click here for additional data file. Supplemental material, MSJ918978_supplemental_material for Cortical axonal loss is associated with both gray matter demyelination and white matter tract pathology in progressive multiple sclerosis: Evidence from a combined MRI-histopathology study by Svenja Kiljan, Paolo Preziosa, Laura E Jonkman, Wilma DJ van de Berg, Jos Twisk, Petra JW Pouwels, Geert J Schenk, Maria A Rocca, Massimo Filippi, Jeroen JG Geurts and Martijn D Steenwijk in Multiple Sclerosis Journal

## References

[1] Faissner S Plemel JR Gold R, et al Progressive multiple sclerosis: From pathophysiology to therapeutic strategies. Nat Rev Drug Discov 2019; 18(12): 905–922. 31399729 10.1038/s41573-019-0035-2

[2] Trapp BD Ransohoff R Rudick R. Axonal pathology in multiple sclerosis: Relationship to neurologic disability. Curr Opin Neurol 1999; 12(3): 295–302. 10499174 10.1097/00019052-199906000-00008

[3] Popescu V Klaver R Voorn P, et al What drives MRI-measured cortical atrophy in multiple sclerosis. Mult Scler 2015; 21(10): 1280–1290. 25583833 10.1177/1352458514562440

[4] Fisher E Lee JC Nakamura K, et al Gray matter atrophy in multiple sclerosis: A longitudinal study. Ann Neurol 2008; 64: 255–265. 18661561 10.1002/ana.21436

[5] Rocca MA Comi G Filippi M. The Role of T1-Weighted Derived Measures of Neurodegeneration for Assessing Disability Progression in Multiple Sclerosis. Front Neurol 2017; 8: 433. 28928705 10.3389/fneur.2017.00433 PMC5591328

[6] Eijlers AJC van Geest Q Dekker I, et al Predicting cognitive decline in multiple sclerosis: A 5-year follow-up study. Brain 2018; 141(9): 2605–2618. 30169585 10.1093/brain/awy202

[7] Calabrese M Magliozzi R Ciccarelli O, et al Exploring the origins of grey matter damage in multiple sclerosis. Nat Rev Neurosci 2015; 16(3): 147–158. 25697158 10.1038/nrn3900

[8] Bodini B Chard D Altmann DR, et al White and gray matter damage in primary progressive MS: The chicken or the egg? Neurology 2016; 86: 170–176. 26674332 10.1212/WNL.0000000000002237 PMC4731689

[9] Haider L Zrzavy T Hametner S, et al The topograpy of demyelination and neurodegeneration in the multiple sclerosis brain. Brain 2016; 139(Pt 3): 807–815. 26912645 10.1093/brain/awv398 PMC4766379

[10] Steenwijk MD Daams M Pouwels PJ, et al Unraveling the relationship between regional gray matter atrophy and pathology in connected white matter tracts in long-standing multiple sclerosis. Hum Brain Mapp 2015; 36(5): 1796–1807. 25627545 10.1002/hbm.22738 PMC6869234

[11] Magliozzi R Reynolds R Calabrese M. MRI of cortical lesions and its use in studying their role in MS pathogenesis and disease course. Brain Pathol 2018; 28(5): 735–742. 30020563 10.1111/bpa.12642 PMC8028295

[12] Seewann A Kooi EJ Roosendaal SD, et al Translating pathology in multiple sclerosis: The combination of postmortem imaging, histopathology and clinical findings. Acta Neurol Scand 2009; 119(6): 349–355. 19254283 10.1111/j.1600-0404.2008.01137.x

[13] Jonkman LE Graaf YG Bulk M, et al Normal Aging Brain Collection Amsterdam (NABCA): A comprehensive collection of postmortem high-field imaging, neuropathological and morphometric datasets of non-neurological controls. Neuroimage Clin 2019; 22: 101698. 10.1016/j.nicl.2019.101698 PMC6360607 30711684

[14] Preziosa P Kiljan S Steenwijk MD, et al Axonal degeneration as substrate of fractional anisotropy abnormalities in multiple sclerosis cortex. Brain 2019; 42: 1921–1937. 10.1093/brain/awz143 31168614

[15] Schindelin J Arganda-Carreras I Frise E, et al Fiji: An open-source platform for biological-image analysis. Nat Meth 2012; 9(7): 676–682. 10.1038/nmeth.2019 PMC3855844 22743772

[16] Jenkinson M Beckmann CF Behrens TE, et al FSL. Neuroimage 2012; 62: 782–790. 21979382 10.1016/j.neuroimage.2011.09.015

[17] Daams M Steenwijk MD Wattjes MP, et al Unraveling the neuroimaging predictors for motor dysfunction in long-standing multiple sclerosis. Neurology 2015; 85(3): 248–255. 26115736 10.1212/WNL.0000000000001756

[18] Evangelou N Esiri MM Smith S, et al Quantitative pathological evidence for axonal loss in normal appearing white matter in multiple sclerosis. Ann Neurol 2000; 47(3): 391–395. 10716264

[19] Klaver R Popescu V Voorn P, et al Neuronal and axonal loss in normal-appearing gray matter and subpial lesions in multiple sclerosis. J Neuropathol Exp Neurol 2015; 74(5): 453–458. 25853695 10.1097/NEN.0000000000000189

[20] Trapp BD Peterson J Ransohoff RM, et al Axonal transection in the lesions of multiple sclerosis. N Engl J Med 1998; 338(5): 278–285. 9445407 10.1056/NEJM199801293380502

[21] Chard DT Griffin CM McLean MA, et al Brain metabolite changes in cortical grey and normal-appearing white matter in clinically early relapsing-remitting multiple sclerosis. Brain 2002; 125(Pt 10): 2342–2352. 12244090 10.1093/brain/awf240

[22] Fu L Matthews PM De Stefano N, et al Imaging axonal damage of normal-appearing white matter in multiple sclerosis. Brain 1998; 121(Pt 1): 103–113. 9549491 10.1093/brain/121.1.103

[23] Carassiti D Altmann DR Petrova N, et al Neuronal loss, demyelination and volume change in the multiple sclerosis neocortex. Neuropathol Appl Neurobiol 2018; 44: 377–390. 28419506 10.1111/nan.12405

[24] Peterson JW Bo L Mork S, et al Transected neurites, apoptotic neurons, and reduced inflammation in cortical multiple sclerosis lesions. Ann Neurol 2001; 50(3): 389–400. 11558796 10.1002/ana.1123

[25] Wegner C Esiri MM Chance SA, et al Neocortical neuronal, synaptic, and glial loss in multiple sclerosis. Neurology 2006; 67(6): 960–967. 17000961 10.1212/01.wnl.0000237551.26858.39

[26] Jonkman LE Klaver R Fleysher L, et al The substrate of increased cortical FA in MS: A 7T post-mortem MRI and histopathology study. Mult Scler 2016; 22(14): 1804–1811. 26945031 10.1177/1352458516635290

[27] Budde MD Xie M Cross AH, et al Axial diffusivity is the primary correlate of axonal injury in the experimental autoimmune encephalomyelitis spinal cord: A quantitative pixelwise analysis. J Neurosci 2009; 29(9): 2805–2813. 19261876 10.1523/JNEUROSCI.4605-08.2009 PMC2673458

[28] Schmierer K Wheeler-Kingshott CA Boulby PA, et al Diffusion tensor imaging of post mortem multiple sclerosis brain. Neuroimage 2007; 35(2): 467–477. 17258908 10.1016/j.neuroimage.2006.12.010 PMC1892244

[29] Klawiter EC Schmidt RE Trinkaus K, et al Radial diffusivity predicts demyelination in ex vivo multiple sclerosis spinal cords. Neuroimage 2011; 55(4): 1454–1460. 21238597 10.1016/j.neuroimage.2011.01.007 PMC3062747

[30] Wheeler-Kingshott CA Cercignani M. About “axial” and “radial” diffusivities. Magn Reson Med 2009; 61: 1255–1260. 19253405 10.1002/mrm.21965

[31] Kolasinski J Stagg CJ Chance SA, et al A combined post-mortem magnetic resonance imaging and quantitative histological study of multiple sclerosis pathology. Brain 2012; 135(Pt 10): 2938–2951. 23065787 10.1093/brain/aws242 PMC3470716

[32] Mesaros S Rocca MA Kacar K, et al Diffusion tensor MRI tractography and cognitive impairment in multiple sclerosis. Neurology 2012; 78(13): 969–975. 22377806 10.1212/WNL.0b013e31824d5859

[33] Van Waesberghe JH Kamphorst W De Groot CJ, et al Axonal loss in multiple sclerosis lesions: Magnetic resonance imaging insights into substrates of disability. Ann Neurol 1999; 46(5): 747–754. 10553992 10.1002/1531-8249(199911)46:5<747::aid-ana10>3.3.co;2-w

[34] Boon BDC Pouwels PJW Jonkman LE, et al Can post-mortem MRI be used as a proxy for in-vivo? A case study. Brain Commun 2019; 1: fcz030. 10.1093/braincomms/fcz030 PMC7425311 32954270

