# Supplementary material for: Cortical axonal loss is associated with both gray matter demyelination and white matter tract pathology in progressive multiple sclerosis: Evidence from a combined MRI-histopathology study
Source: Mult Scler. 2020 May 11;27(3):380–90. doi: 10.1177/1352458520918978 (PMC7897796; doi:10.1177/1352458520918978)
Supplement: MSJ918978_supplemental_material – Supplemental material for Cortical axonal loss is associated with both gray matter demyelination and white matter tract pathology in progressive multiple sclerosis: Evidence from a combined MRI-histopathology study [file MSJ918978_supplemental_material.pdf]

Subjects and data collection pipeline

In this study 16 cases with clinically definite and neuro-pathologically verified MS (5 males, 11 females, mean age=64±13y, all PMS) and 10 non-neurological controls (5 males, 5 females, mean age=72±5y) were included shortly after death (mean *post-mortem* delay until autopsy (PMD) in MS: 08:11±1:00h; mean PMD in non-neurological controls: 10:05±3:05h; Table 1). A Mann-Whitney U test was performed to establish group differences in age and PMD, while a Fisher exact test was performed to test group differences in gender. MS patients and controls did not significantly differ on sex, age and PMD (p=0.425; p=0.140; p=0.162, respectively).

Immunohistochemistry and Bielschowksy staining

Paraffin sections were cut and collected on superfrost plus glass slides (VWR International, Leuven, Belgium), dried vertically overnight at room temperature (RT) and subsequently dried horizontally for three hours at 45°C and then overnight at 37°C. Prior to staining, sections were deparaffinized in xylene substitute (Sigma-Aldrich, Zwijndrecht, the Netherlands) and rehydrated through a series of ethanol (EtOH) and rinsed in miliQ water.

Immunostaining was performed of cortical myelin (proteolipidprotein; PLP; 1:500; MCA839G; Bio-Rad Laboratories B.V., Veenendaal, The Netherlands), microglia (ionized calcium-binding adapter molecule 1; Iba-1; 1:2000; Abcam, Cambridge, MA) and neurons (neuronal nuclei; NeuN; 1:1000; Novus Biologicals, Oakville, Canada). Also a silver staining was performed to visualize axons (Bielschowksy impregnation).

Quantification of neuro-axonal degeneration and cortical pathology

Per section two regions of interest (ROIs) of approximately 8 mm2 each, were randomly selected in six-layered and non-curved parts of the cortex. Afterwards ROIs were classified as containing lesioned or cortical NAGM by an experienced rater (S.K.).

Images of Bielschowsky staining were acquired using a Leica DM/RBE photomicroscope (Leica, Heidelberg, Germany) at 200x magnification. Images were stitched to obtain one image per ROI including all cortical layers. Analyses of axonal density was performed manually in ImageJ/Fiji (version 1.52a, https://imagej.net/Fiji).[1] Vertical lines running from the pial surface to the WM were superimposed on every image *i.e.* ROI. Axonal density was obtained by counting the intersection of axons with either a vertical line or with horizontal lines that were computed along the entire length of the vertical line every 500µm, to obtain a density measure without bias of fiber orientation (Figure 1B). Axons intersecting with both lines or with one line twice were only counted once. Axonal density was expressed as axon number per mm.

Neuronal density and volume were quantified using a Leica DMR microscope (Leica Microsystems, Wetzlar, Germany) in combination with stereoinvestigator software (MBF Bioscience, Williston, VT; Figure 1D). The optical fractionator tool was used to obtain density measures of neurons unbiased for orientation, shape and size in thick sections.[2] The nucleus top was used as a unique point for counting, while sampling in a random systematic manner throughout the ROIs in 3D at 630x magnification (oil lens). Per section, 103±30 $NeuN^+$ cells were counted on average, the coefficient of error was 0.10±0.02 (Schmitz and Hof).[3] The nucleator tool was used to quantify neuronal volume while sampling with the optical fractionator, using the nucleolus as a unique point.[4]

Microglia density was quantified using a Leica Ctr 5000 microscope (Leica Microsystems, Wetzlar, Germany) with a Nuance spectral imaging device and associated Nuance spectral imaging software (Nuance version 3.0.2, Caliper Kife Sciences, Inc, a Perkin Elmer Company, Hopkinton, MA). Separate images were acquired of cortical layers 1 and 2, layer 3 and layer 5 and 6, at 200x magnification. To obtain robust measures of microglia density two images were acquired per cortical layer and results were averaged. Spectral information of Iba-1 immunostaining was used to compute a corresponding mask and microglia density was expressed as percentage of stained area of the image area.

Myelin density was quantified within ROIs using images of PLP stained sections that were acquired using a Leica DM/RBE photomicroscope (Leica, Heidelberg, Germany) at 50x magnification. Images were stitched to obtain a single image of the entire section. ImageJ was used to superimpose the ROIs onto the images. A mask of the myelin staining was computed and myelin density was expressed as percentage of stained area of the ROI area.

Quantification of white matter pathology in connected tracts

In each subject, MRI of the brain *in situ* was acquired using a 3T whole-body scanner (Signa HDxt; General Electrics, Milwaukee, Wisconsin, USA) and an eight-channel phased-array head coil. The protocol included a three-dimensional T1-weighted fast spoiled gradient-echo sequence for volumetric analysis (repetition time 7.8ms, echo time 3.0ms, inversion time 450ms, slice thickness 1mm, in-plane resolution 0.9x0.9 mm^2), a three-dimensional fluid-attenuated inversion-recovery sequence for WML detection (repetition time 8000ms, echo time 125ms, inversion time 2350ms, slice thickness 1.2mm, in-plane resolution 0.98x0.98 mm^2), a diffusion-tensor imaging sequence (30 volumes with non-collinear diffusion gradients, i.e. 30 directions, [b=1000sec/mm^2] and five volumes without diffusion weighting) for tractography (repetition time 13000ms, echo time 91ms, slice thickness 2.4mm, in-plane resolution 2.0x2.0 mm^2).

A group-level probabilistic WM tract atlas, based on an *in vivo* healthy control dataset (23 males, 37 females, mean age=50±7), was finally constructed by non-linear co-registration of each subjects' binarized tracts to Montreal Neurological Institute (MNI) template space. The resulting atlas was used

to quantify WM pathology in the donors. WML were automatically segmented from the FLAIR and T1 images using an in house developed algorithm followed by manual adjustments of an experienced rater (P.P.).[5] WML filling was performed on T1-weighted images to reduce the impact of hypo-intense WML on subsequent image processing.[6] Non-linear registration was performed to transform the probabilistic WM atlas and WML mask into subject-wise diffusion space.

1. Schindelin J, Arganda-Carreras I, Frise E, et al. Fiji: an open-source platform for biological-image analysis. Nat Methods 2012;9:676-682.
2. Gundersen HJ, Jensen EB. The efficiency of systematic sampling in stereology and its prediction. J Microsc 1987;147:229-263.
3. Schmitz C, Hof PR. Design-based stereology in neuroscience. Neuroscience 2005;130:813-831.
4. Gundersen HJ. The nucleator. J Microsc 1988;151:3-21.
5. Steenwijk M, Daams M, Barkhof F, Pouwels P, Geurts J. Multi-view convolutional neural networks using batch normalization outperform human raters during automatic white matter lesion segmentation. ECTRIMS online library http://onlinelibraryectrims-congresseu/ectrims/2017/ACTRIMSECTRIMS2017/200729/martijndsteenwijkmultiviewconvolutionalneuralnetworksusingbatchhtml 2017.
6. Chard DT, Jackson JS, Miller DH, Wheeler-Kingshott CA. Reducing the impact of white matter lesions on automated measures of brain gray and white matter volumes. J Magn Reson Imaging 2010;32:223-228.
